# Association between cup orientation and low back pain after total hip arthroplasty in patients with osteonecrosis of the femoral head

**DOI:** 10.1186/s12891-023-07139-6

**Published:** 2024-01-02

**Authors:** Tong Li, Yifei Li, Weiguo Wang

**Affiliations:** 1 Department of Orthopedics, China-Japan Friendship Hospital, Beijing, China; 2 15th Department, Plastic Surgery Hospital, Chinese Academy of Medical Sciences and Peking Union Medical College, Beijing, China

**Keywords:** Hip-spine syndrome, Bilateral total hip arthroplasty (THA), Postoperative low back pain (LBP), Cup orientation, Ante-inclination (AI)

## Abstract

**Background:**

Postoperative low back pain (LBP) following total hip arthroplasty (THA) is classified as secondary hip-spine syndrome. The purpose of this study was to explore the correlations between cup orientation of THA and postoperative LBP in patients with osteonecrosis of the femoral head (ONFH).

**Methods:**

A retrospective cohort study included 364 ONFH patients who underwent bilateral THA between January 2011 and December 2020. Among them, 53 patients (14.6%) experienced postoperative LBP at the end of follow-up and were designated as pain group (PG). A control group (CG) consisting of 106 patients with similar age, sex, and body mass index (BMI) to those in the PG was selected. Postoperative LBP in the PG was assessed using the visual analogue scale (VAS). Demographic data, clinical information, and radiographic criteria were evaluated as potential predictors of LBP.

**Results:**

Patients in PG (mean age, 47.3 years [range, 27 to 75 years]; 42 [79%] male) had a mean VAS score of 4.6 (range, 1 to 9) compared with 0 for the patients in CG (mean age, 47.6 years [range, 19 to 77 years]; 84 [79%] male). There were no significant differences in clinical data between the two groups (*p* > 0.05). Preoperative radiographic variables also showed no significant differences between the PG and CG (*p* > 0.05). However, the postoperative inclination, anteversion, and standing ante-inclination (AI) were significantly lower in the PG compared to the CG, whereas the sitting and standing sacral slope (SS) were significantly higher (*p* < 0.05). Moreover, the variations in standing AI, standing and sitting pelvic tilt (PT) were significantly lower in the PG compared to the CG, while the variations in standing and sitting SS and lumbar lordosis (LL) were significantly higher (*p* < 0.05). The variation in standing AI in the PG showed a significantly correlation with the variation of standing SS, standing PT, and LL (*p* < 0.05).

**Conclusion:**

Postoperative LBP in ONFH patients after bilateral THA is significantly associated with the intraoperative cup orientation. The variation in standing AI is correlated with the variations in standing SS, standing PT, and LL, potentially contributing to the development of postoperative LBP.

## Background

The concept of hip-spine syndrome, initially proposed and categorized by Offierski and MacNab in 1983, refers to the coexistence of pathologies affecting both the hip and the spine [1]. This syndrome is classified into three categories, namely simple, complex, and secondary hip-spine syndrome, with the latter characterizing a scenario wherein interlinked pathological processes that mutually exacerbate each other. Recently, there has been considerable focus on spinopelvic sagittal alignment, leading to numerous studies investigating spinal alignment within the context of hip disorders [2–4].

Total hip arthroplasty (THA) stands as a widely conducted and notably efficacious surgical procedure intended for the management of advanced osteonecrosis of femoral head (ONFH), particularly in cases accompanied by femoral head collapse and secondary acetabular change [5]. The application of THA in patients afflicted by ONFH, comprising 3–12% of all primary THA cases, poses distinctive challenges, as this demographic encompasses a younger age group and displays inferior clinical outcomes compared to individuals with osteoarthritis [6]. In light of these considerations, it is crucial for patients undergoing THA due to ONFH to prioritize not only the augmentation of long-term prosthetic durability but also the mitigation of potential postoperative complications.

Notably, postoperative low back pain (LBP) has been identified as complication of THA and can be classified as secondary hip-spine syndrome. In support of this, a study by Parvizi J et al. highlighted that out of 174 patients without LBP prior to THA, 35 patients developed LBP subsequent to the surgery. In terms of etiology, aside from patients diagnosed with lumbar degeneration and disc herniation, a subset exhibited negative findings in their spine evaluations [7]. Patients diagnosed with ONFH are typically younger, exhibit less spinal degeneration, and consequently have a lower risk of experiencing LBP resulting from spontaneous spinal degeneration after THA compared to those with hip osteoarthritis. Postoperative LBP in these cases may be likely attributed to alterations in the sagittal alignment of the spine after hip surgery. The dynamic interplay between the lumbosacral joint and hip joint can be likened to the operation of two hinges, with the pelvis functioning as a “gear” to connect them. Thus, we proposed the following hypothesis: deviations in the orientation of the acetabular cup can potentially trigger compensatory adjustments within the lumbosacral joint, aimed at sustaining sagittal balance, thereby potentially leading to secondary LBP.

While early studies primarily focused on the improvement of preoperative LBP and spine function through THA by altering sagittal spinopelvic alignment, scant attention has been directed towards the exploration of new occurrences of LBP after THA [8–11]. The objective of this study was to comprehensively investigate the correlations between cup orientation in THA and the subsequent emergence of postoperative LBP in patients with ONFH.

## Methods

**Fig. 1:**
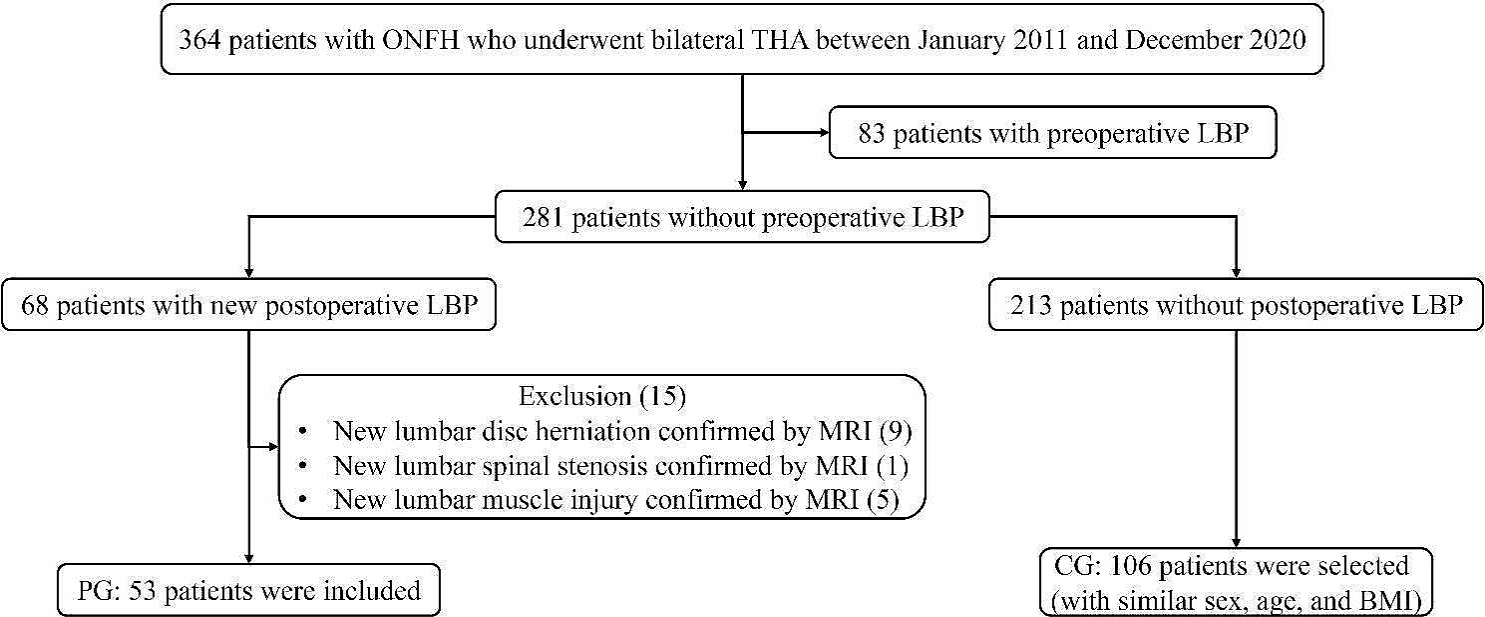
Study flowchart

A consecutive series of 364 patients with ONFH who underwent bilateral THA at our hospital between January 2011 and December 2020 were retrospectively recruited for this study. Specifically, our study focused on a subset of 281 patients who were devoid of LBP or notable lumbar degenerative changes on X-ray prior to the surgical procedure. Subsequent follow-up assessments unveiled that a total of 68 individuals from this subset encountered new occurrences of LBP within a 2-year interval subsequent to the surgery (Fig. 1).

The inclusion criteria for the pain group (PG) were as follows: (1) confirmation of bilateral ONFH through preoperative hip X-ray, (2) absence of LBP symptoms prior to THA, (3) no history of lumbar disease or prior lumbar surgery before THA; (4) development of new LBP within 2 years after THA, and (5) exclusion of new lumbar disc herniation, lumbar spinal stenosis, lumbar muscle strain, infection, tumor through magnetic resonance imaging (MRI). The exclusion criteria for the PG were as follows: (1) confirmation of hip osteoarthritis, developmental dysplasia of the hip, or femoral neck fracture through preoperative hip X-ray, (2) preoperative LBP symptoms or a history of low back disease or surgery, and (3) new LBP symptoms caused by postoperative lumbar disc herniation, lumbar spinal stenosis, lumbar muscle injury, infection, tumor, confirmed by MRI. Ultimately, a total of 53 patients meeting the established criteria were included in the PG (Fig. 1).

Additionally, to facilitate comparative analysis, a control group (CG) was formed, comprising 106 patients matched for age, sex, and body mass index (BMI) to those of the 53 patients who experienced postoperative LBP. These control participants were selected from the remaining patient pool devoid of preoperative LBP (Fig. 1).

### Surgical technique

The patient was positioned on their side for the posterior approach. Starting below the greater trochanter, the incision curved upward toward the back of the pelvis. The surgeon cut through the outer layer over the gluteus muscle and separated it to access the short external rotator muscles. A Charnley retractor was utilized to retract the gluteus muscle while ensuring the sciatic nerve was protected. The short external rotators and piriformis muscles were incised at their attachment points on the greater trochanter and marked for later repair. A pendulum saw was used to dissect the bone, removing the femoral head with a head extractor. Access was then gained to the acetabulum and proximal femur. Careful placement of Hohmann retractors around the acetabulum provided sufficient exposure. The femur was retracted anteriorly to properly expose the acetabulum for restoration of acetabular anteversion. A posterior retractor assisted in retracting the posterior joint capsule for acetabular visualization. An acetabulum file was used to refine the acetabulum size. Soft tissue landmarks were employed during acetabular preparation to verify anteversion and inclination, ensuring accurate placement of the acetabular cup. The target anteversion was set at 15°, while the inclination aimed for 40°. The cementless Pinnacle acetabular cup (DePuy Synthes, Warsaw, IN, USA) with a ceramic liner was employed. The proximal femur was exposed with the leg internally rotated, flexed, and slightly adducted, aligning the long axis of the tibia vertically. A blunt bone skid aided in elevating the femur for improved exposure. The femoral bone marrow cavity was extended to an appropriate size, a fitting cementless Corail femoral stem (DePuy Synthes, Warsaw, IN, USA) was chosen and implanted, and subsequently, the hip joint was restored to its original position. Finally, the short external rotators and posterior capsule were repaired through transosseous bone tunnels in the proximal femur.

### Postoperative rehabilitation protocol

1. Day of surgery to discharge: Physical therapy began either on the surgery day or afterward, focusing on exercises to prevent blood clots, enhance circulation, and restore basic mobility.
2. Immediate postoperative period (1–6 weeks): Patients started with support aids like walkers, crutches, or canes, gradually extending their walking distance. Pain and swelling post-surgery were managed using medications and modalities like ice packs. Gentle exercises were introduced to enhance joint mobility.
3. Intermediate postoperative period (6 weeks − 3 months): There was a gradual shift toward full weight-bearing. Exercises were introduced to bolster the hip and lower limb muscles, emphasizing stability and support. Balance and coordination exercises were incorporated to improve mobility and decrease the risk of falls.
4. Advanced postoperative period (3 months and beyond): More advanced exercises were introduced to further enhance hip function and endurance.

### Data collection

Data pertaining to patients’ clinical characteristics, including age, gender, BMI, type of surgery (simultaneous or staged), etiology, Association Research Circulation Osseous (ARCO) classification, disease duration, and Harris hip score, were collected. In cases where postoperative LBP was reported, the severity was evaluated using a Visual Analog Scale (VAS) score ranging from 0 to 10.

**Fig. 2:**
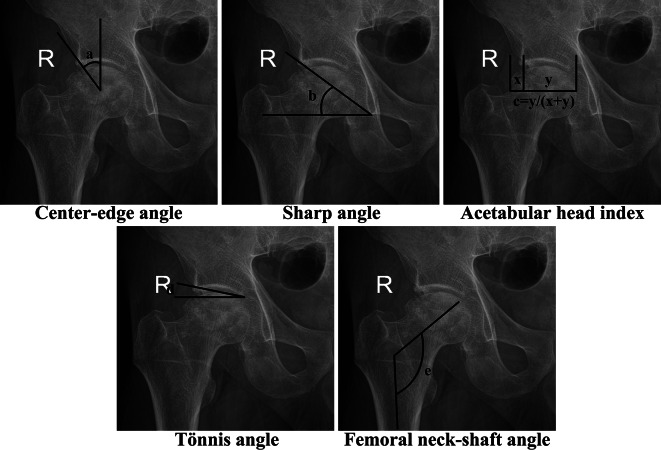
Measurements of the anatomical parameters of the hip joint. (**a**). Center-edge angle; (**b**). Sharp angle; (**c**). Acetabular head index; (**d**). Tönnis angle; (**e**). Femoral neck-shaft angle

**Fig. 3:**
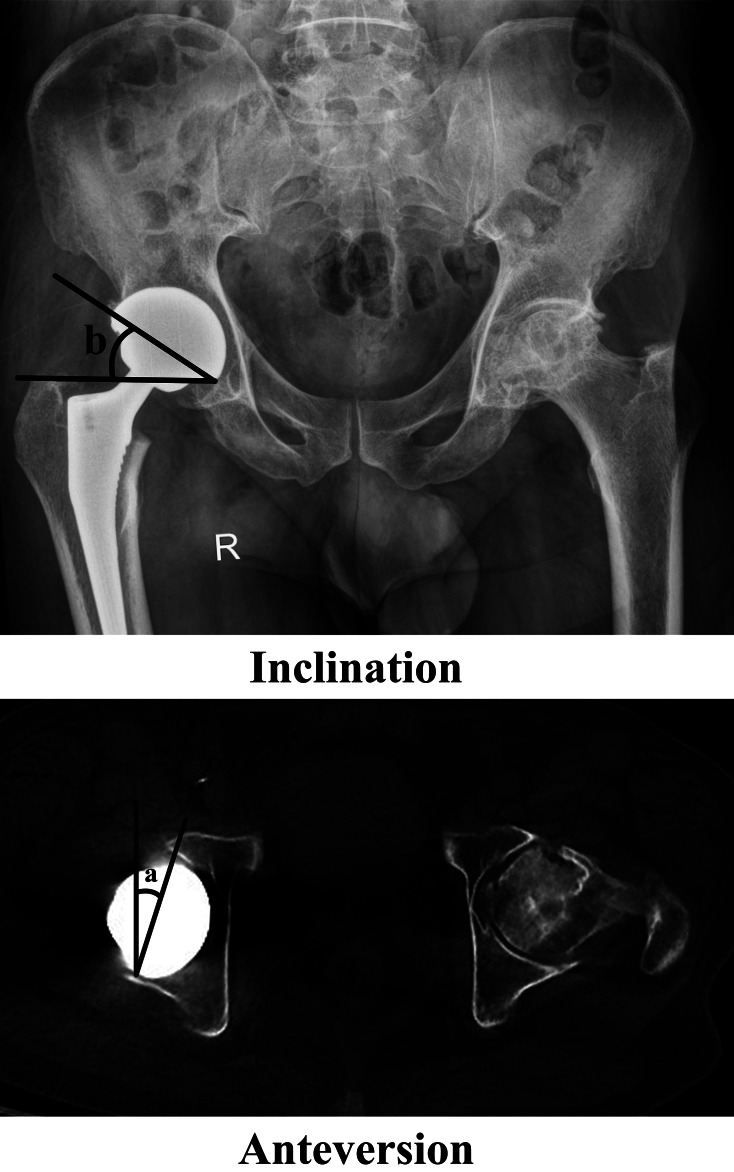
Measurements of the inclination and anteversion. (**a**). Inclination; (**b**). Anteversion

**Fig. 4:**
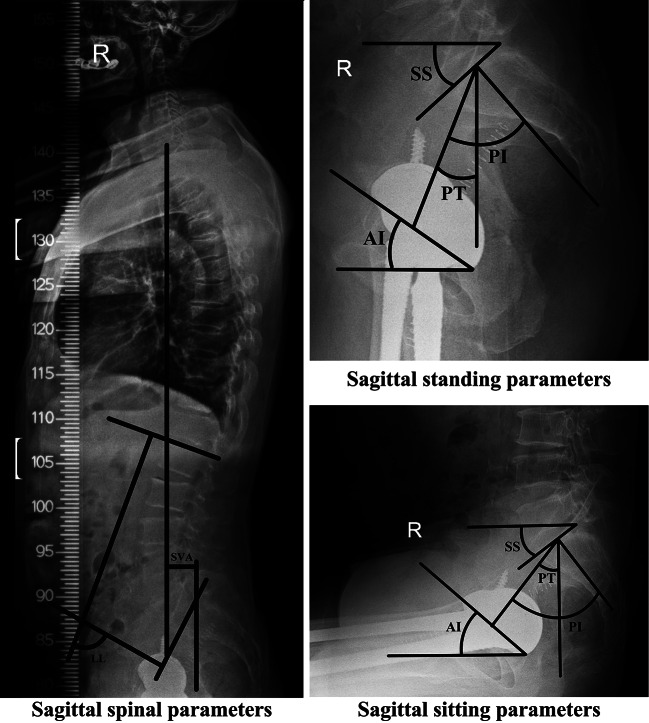
Measurements of the sagittal spinopelvic parameters

Furthermore, both pre- and post-surgery radiographic evaluations were conducted for all participants. Within the PG, imaging assessments were specifically administered upon the onset of postoperative LBP during follow-up, while in the CG, these assessments were carried out 2 years post-surgery. For each patient, the radiography data comprised anteroposterior (AP) radiographs of the hip and pelvis, standing and sitting lateral radiographs of the hip and pelvis, lateral radiographs of the entire spine, pelvic computed tomography (CT) scans, and lumbar MRI. The AP hip views were employed preoperatively to evaluate the anatomical parameters of the hip joint, including the center-edge (CE) angle, Sharp angle, acetabular head index (AHI), Tönnis angle, and femoral neck-shaft angle (Fig. 2). The AP radiographs and CT scans of the pelvis enabled the assessment of the acetabulum’s inclination and anteversion both pre- and post-surgery (Fig. 3). Inclination denotes the angle between the opening of the acetabulum (acetabular cup) and the transverse axis of the pelvis, while anteversion signifies the angle between the opening plane of the acetabulum (acetabular cup) and the sagittal plane. The lateral radiographs of the hip were utilized to measure the ante-inclination (AI) [12] of the acetabulum pre-surgery and the AI of the cup post-surgery (Fig. 4). All hip parameters were measured bilaterally. The anatomical parameters of pelvis and spine, including pelvic tilt (PT), pelvic incidence (PI), sacrum slop (SS), lumbar lordosis (LL) and sagittal vertical axis (SVA), were evaluated using lateral radiographs of the pelvis and spine obtained before and after surgery (Fig. 4). The digital images were stored and retrieved for measurement using the Carestream Vue HIMS system. To mitigate potential systematic bias, the measurements were independently conducted by two researchers and then averaged.

### Statistical analysis

The data were analyzed using SPSS version 26.0. The propensity score matching was employed to achieve a 1:2 matching ratio. A comparative assessment of each parameter was conducted pre- and post-surgery, with the change denoted as Δ. A single-measure (2-way random) intraclass correlation coefficient was used to quantify interobserver reliability (values of > 0.75 indicate satisfactory reliability). Initially, the Shapiro-Wilk test was utilized to assess to the normal distribution of the measurement data, while the Levene test was conducted to assess the homogeneity of variance within the data. Data meeting the criteria for both normal distribution and variance homogeneity, designated as $\bar{x}$ ± s, were subject to comparison using t-test and ANOVA. In cases where the data did not meet these criteria, signified as M(Q_1_, Q_3_) due to non-normal distribution or variance heterogeneity, group comparisons were executed using the Mann-Whitney U test. Statistical descriptions of counting data were performed through frequency analysis, with comparisons made using the Chi-square test. In cases where postoperative LBP was reported, the Spearman correlation coefficient was utilized to explore the associations between the variation of hip parameters and the variation of sagittal spinopelvic parameters. A significance threshold of α < 0.05 was established to determine statistical significance.

## Results

Baseline demographic characteristics, encompassing factors such as simultaneous operation, etiology, ARCO classification, disease duration, Harris hip score, and follow-up period, exhibited no statistically significant differences between patients who experienced postoperative LBP and those who did not (*p* > 0.05, Table 1). Intraclass correlations were shown in Table 2. Similarly, no significant differences were observed in preoperative radiographic variables between the two groups (*p* > 0.05, Table 3).

**Table 1:** Basic characteristics of patients in the PG compared with CG

Variable			PG (n=53)	CG (n=106)	*p* value	95% CI for difference in means
Gender (male/female)			42/11	84/22	1	
Age			47.3±11.8	47.6±11.3	0.872	-4.13 to 3.51
BMI			25.3±3.3	25.3±3.9	0.943	-1.27 to 1.18
Simultaneous surgery (%)			25	34	0.063	
Etiology					0.961	
	Alcohol		19	38		
	Corticosteroid		14	30		
	Idiopathic		20	38		
ARCO classification						
Left		III	21	43	0.909	
		IV	32	63		
Right		III	26	49	0.736	
		IV	27	57		
Disease duration (month)			57.0±78.3	69.1±69.5	0.323	-36.18 to 12.01
Harris hip score						
		Left	58.7±11.8	59.2±12.2	0.845	-5.34 to 4.38
		Right	58.8±15.8	61.1±12.7	0.419	-7.97 to 3.34
Follow-up period (month)			37.8±7.3	36.7±5.9	0.322	-1.05 to 3.19

**Table 2:** Intraclass correlation coefficient for radiographic measurements

Variable		Intraclass correlation	95% CI	*p* value
Center-Edge (CE) angle (°)	Left	0.909	0.878 to 0.933	< 0.001
	Right	0.968	0.957 to 0.977	< 0.001
Sharp angle (°)	Left	0.949	0.931 to 0.963	< 0.001
	Right	0.921	0.894 to 0.941	< 0.001
Acetabular coverage (%)	Left	0.917	0.888 to 0.938	< 0.001
	Right	0.955	0.938 to 0.967	< 0.001
Tönnis angle (°)	Left	0.855	0.807 to 0.892	< 0.001
	Right	0.919	0.891 to 0.940	< 0.001
Femoral neck-shaft angle (°)	Left	0.959	0.944 to 0.970	< 0.001
	Right	0.943	0.922 to 0.958	< 0.001
Inclination (°)				
Preoperative	Left	0.992	0.989 to 0.994	< 0.001
	Right	0.991	0.988 to 0.994	< 0.001
Postoperative	Left	0.992	0.990 to 0.994	< 0.001
	Right	0.988	0.984 to 0.991	< 0.001
Anteversion (°)				
Preoperative	Left	0.975	0.965 to 0.981	< 0.001
	Right	0.979	0.972 to 0.985	< 0.001
Postoperative	Left	0.966	0.954 to 0.975	< 0.001
	Right	0.965	0.952 to 0.974	< 0.001
Ante-Inclination (AI) (standing) (°)				
Preoperative	Left	0.958	0.942 to 0.970	< 0.001
	Right	0.969	0.958 to 0.977	< 0.001
Postoperative	Left	0.960	0.945 to 0.970	< 0.001
	Right	0.965	0.953 to 0.975	< 0.001
Ante-Inclination (AI) (sitting) (°)				
Preoperative	Left	0.976	0.967 to 0.982	< 0.001
	Right	0.975	0.966 to 0.982	< 0.001
Postoperative	Left	0.972	0.962 to 0.979	< 0.001
	Right	0.971	0.961 to 0.979	< 0.001
Pelvic tilt (PT) (°)				
Preoperative	standing	0.984	0.979 to 0.989	< 0.001
	sitting	0.986	0.981 to 0.990	< 0.001
Postoperative	standing	0.983	0.977 to 0.988	< 0.001
	sitting	0.987	0.983 to 0.991	< 0.001
Pelvic incidence (PI) (°)		0.981	0.974 to 0.986	< 0.001
Sacral slope (SS) (°)				
Preoperative	standing	0.980	0.973 to 0.985	< 0.001
	sitting	0.987	0.982 to 0.990	< 0.001
Postoperative	standing	0.985	0.979 to 0.989	< 0.001
	sitting	0.988	0.984 to 0.991	< 0.001
Lumbar lordosis (LL) (°)				
Preoperative		0.983	0.977 to 0.988	< 0.001
Postoperative		0.985	0.978 to 0.989	< 0.001
Sagittal vertical axis (SVA) (mm)				
Preoperative		0.997	0.996 to 0.998	< 0.001
Postoperative		0.999	0.998 to 0.999	< 0.001

**Table 3:** Comparison of radiographic measurements between patients in the PG and CG

Variable		PG (n=53)			CG (n=106)		
		Preoperative	Postoperative	Variation	Preoperative	Postoperative	Variation
Center-Edge (CE) angle (°)	Left	36(33,38)			36(33,37)		
	Right	36(33,40)			38(35,38)		
Sharp angle (°)	Left	36(35,37)			36(35,38)		
	Right	36(35,38)			36(35,39)		
Acetabular coverage (%)	Left	84(81,86)			84(78,85)		
	Right	83(80,86)			82(78,85)		
Tönnis angle (°)	Left	7(7,8)			8(7,8)		
	Right	7(7,8)			8(7,8)		
Femoral neck-shaft angle (°)	Left	125(121,127)			126(123,132)		
	Right	126(122,128)			126(123,130)		
Inclination (°)	Left	40(38,42)	40(38,42)	-1(-1,0)	41(39,43)	42(38,43)*	0(-1,2)
	Right	40(39,42)	40(38,42)	-1(-2,0)	41(40,43)	42(39,43)*	1(-1,2)
Anteversion (°)	Left	15(14,16)	14(14,16)	-1(-1,1)	15(14,16)	15(14,16)*	1(-1,1)
	Right	15(14,16)	15(14,15)	-1(-1,1)	15(14,16)	15(14,16)*	0(-1,1)
Ante-Inclination (AI) (standing) (°)	Left	34(31,35)	33(30,36)	-1(-2,1)	34(33,36)	35(33,36)*	0(-1,1)*
	Right	33(32,36)	33(30,35)	-1(-2,1)	34(33,36)	35(33,36)*	0(-1,1)*
Ante-Inclination (AI) (sitting) (°)	Left	50(48,54)	51(48,54)	0(-1,1)	52(50,55)	52(49,54)	1(-1,1)
	Right	52(50,54)	50(48,54)	-1(-2,1)	52(50,54)	52(50,55)	0(-1,1)
Pelvic tilt (PT) (standing) (°)		13(9,17)	10(5,15)	-2(-4,0)	13(10,16)	12(9,17)	0(-3,3)*
Pelvic tilt (PT) (sitting) (°)		31(26,36)	29(26,34)	-1(-3,1)	32(27,38)	32(27,36)	0(-2,2)*
(standing PT - sitting PT) (°)		-19(-22,-16)	-20(-23,-17)		-19(-21,-17)	-19(-23,-16)	
Pelvic incidence (PI) (°)		51(47,57)			51(47,58)		
Sacral slope (SS) (standing) (°)		39(36,42)	41(38,44)	2(0,4)	39(36,41)	39(36,41)*	0(-3,3)*
Sacral slope (SS) (sitting) (°)		20(18,22)	22(17,24)	1(-1,3)	19(17,22)	19(17,21)*	0(-2,2)*
(standing SS - sitting SS) (°)		19(16,22)	20(17,23)		19(17,21)	19(16,23)	
Lumbar lordosis (LL) (°)		59(51,66)	60(53,67)	2(-1,4)	57(51,63)	56(50,62)	-1(-2,2)*
Sagittal vertical axis (SVA) (mm)		5(-14,25)	6(-15,24)	0(-1,1)	8(-12,17)	8(-13,18)	0(-1,1)

*Difference from low back pain group with statistical significance. (*p* < 0.05, Mann-Whitney U test)

M(Q1, Q3) denotes the median (M) along with the first quartile (Q1) and the third quartile (Q3) of data

Patients who encountered postoperative LBP had a mean VAS pain score of 4.6 (range, 1 to 9). Among those with postoperative LBP, 13 cases reported LBP in the thoracolumbar region, 21 cases in the lumbar region, and 19 cases in the lumbosacral region.

Upon comparison of the PG and CG in Table 3, the postoperative inclination (Left side: *p* = 0.016; Right side: *p* = 0.023), anteversion (Left side: *p* = 0.032; Right side: *p* = 0.029), and standing AI (Left side: *p* = 0.003; Right side: *p* = 0.001) were significantly lower in the PG, while the standing and sitting SS were significantly higher (standing SS: *p* = 0.001; sitting SS: *p* = 0.015). Furthermore, the variations in standing AI (Left side: *p* = 0.003; Right side: *p* < 0.001), standing and sitting PT (standing PT: *p* = 0.014; sitting PT: *p* = 0.043) were significantly lower in the PG compared to the CG, whereas the variations in standing and sitting SS (standing SS: *p* = 0.017; sitting SS: *p* = 0.047), as well as LL (*p* = 0.008), were significantly higher.

**Fig. 5:**
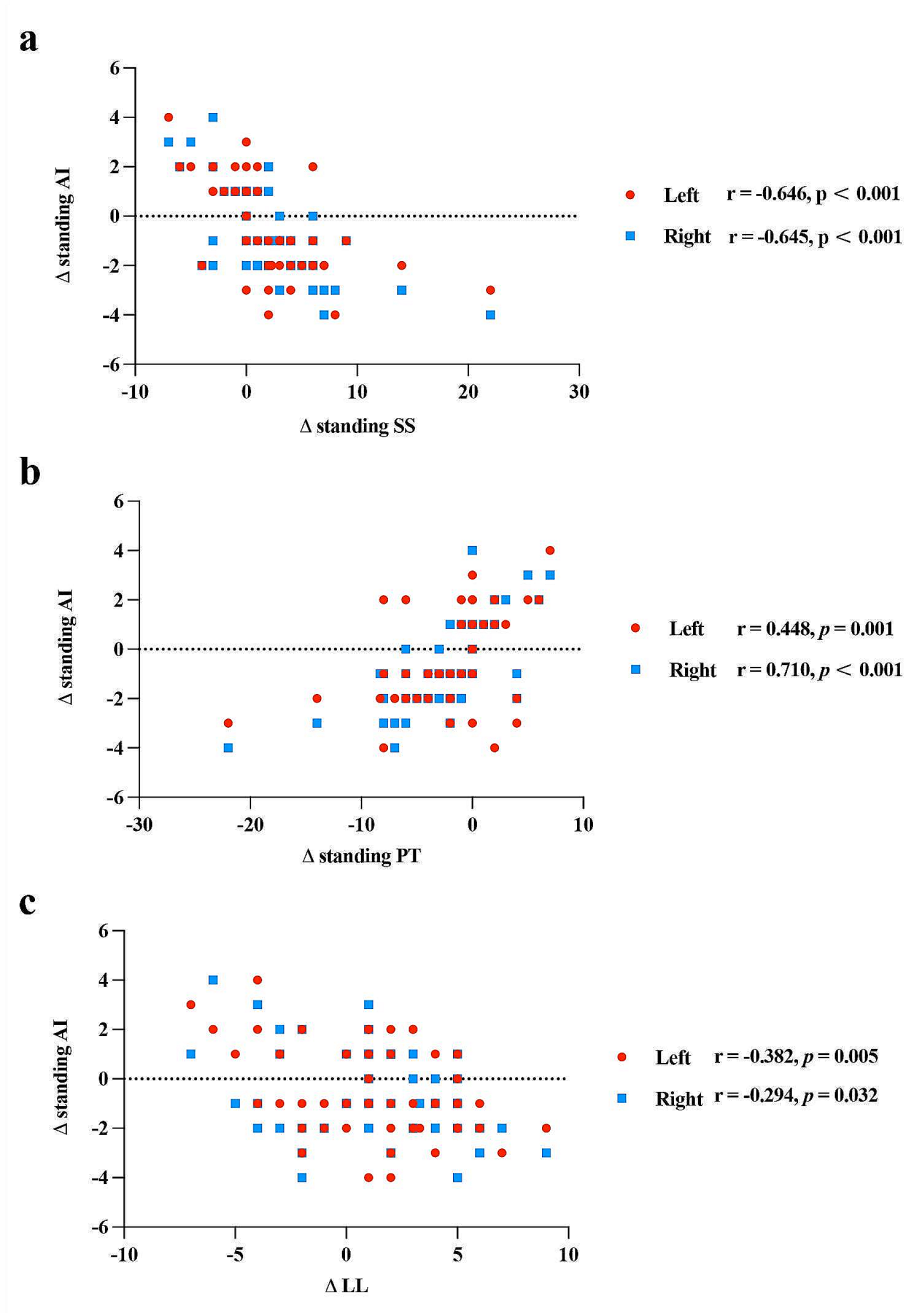
Scatter plots illustrating the correlation between the variations of hip parameters and spinopelvic parameters among patients in the PG. (**a**). A scatter plot illustrating the correlation between Δ standing AI and Δ standing SS. (**b**). A scatter plot illustrating the correlation between Δ standing AI and Δ standing PT. (**c**). A scatter plot illustrating the correlation between Δ standing AI and Δ LL

As evidenced by the data in Table 4, the variation in standing AI before and after surgery in the PG showed a significant correlation with the variation of standing SS, standing PT, and LL (*p* < 0.05). In Fig. 5, there was a negative correlation between the variation in standing SS and standing AI (Left side, r = -0.646, *p* < 0.001; Right side, r = -0.645, *p* < 0.001), a positive correlation between the variation in standing PT and standing AI (Left side, r = 0.488, *p* = 0.001; Right side, r = 0.710, *p* < 0.001), and a negative correlation between the variation in LL and standing AI (Left side, r = -0.382, *p* = 0.005; Right side, -0.294, *p* = 0.032).

**Table 4:** Correlation analysis between the variations of hip and spinopelvic parameters among patients in the PG

			Δ sitting SS	Δ standing SS	Δ sitting PT	Δ standing PT	Δ LL	Δ SVA
Δ Inclination								
	Left	r value	0.026	0.084	-0.065	0.046	-0.049	-0.030
		*p* value	0.856	0.550	0.642	0.742	0.729	0.832
	Right	r value	-0.212	-0.068	0.230	0.068	0.028	0.106
		*p* value	0.127	0.628	0.098	0.631	0.842	0.449
Δ Anteversion								
	Left	r value	-0.068	0.014	0.029	-0.071	-0.046	-0.075
		*p* value	0.630	0.922	0.834	0.615	0.744	0.593
	Right	r value	-0.142	-0.067	0.183	0.024	-0.003	0.330
		*p* value	0.312	0.633	0.190	0.864	0.984	0.815
Δ sitting AI								
	Left	r value	0.113	0.035	-0.074	-0.036	-0.220	0.132
		*p* value	0.420	0.804	0.597	0.796	0.113	0.344
	Right	r value	-0.062	-0.225	0.109	0.251	0.190	0.085
		*p* value	0.660	0.105	0.435	0.069	0.174	0.546
Δ standing AI								
	Left	r value	-0.018	-0.646**	0.004	0.448*	-0.382*	-0.120
		*p* value	0.898	<0.001	0.979	0.001	0.005	0.393
	Right	r value	0.067	-0.645**	-0.066	0.710**	-0.294*	-0.132
		*p* value	0.633	<0.001	0.638	<0.001	0.032	0.345

*Significant at *p* < 0.05, **Significant at *p* < 0.001 (Spearman correlation analysis, two-tailed)

Regarding postoperative complications, hip dislocation was observed in 1(1.9%) case from the PG and 3(2.8%) from the CG due to incorrect posture. Neither the patients from the PG nor the CG experienced periprosthetic fractures or infections during their postoperative follow-up.

## Discussion

Lewinnek et al. proposed the “safe zone” for cup orientation, with an inclination of 40° ± 10° and an anteversion of 15° ± 10°, aiming to reduce dislocation rate [13]. Importantly, the orientation of the acetabular cup during THA is intricately influenced by pelvic tilt [14]. When assessing the correlation between spinopelvic sagittal alignment and acetabular cup orientation, a more appropriate parameter is AI, which takes into account both inclination and anteversion and is sensitive to the changes of pelvic tilt [12]. Previous research has employed AI to investigate the sagittal spinopelvic motion and impingement in THA [15]. Hence, AI offers a more precise evaluation when examining the correlations between spinopelvic sagittal balance and acetabular cup orientation. In this study, we observed significantly lower bilateral inclination, anteversion, standing AI, and its variation in the PG compared to the CG at the last follow-up. These results indicate that the difference of the cup orientation may be a potential cause of postoperative LBP after THA.

Spinopelvic parameters, including the PT, PI, LL, and SVA, have been shown to be correlated with pain and disability [16]. These parameters are used as reference values to determine appropriate realignment procedures [17]. PT reflects the degree of anterior-posterior pelvic tilt and varies with the patient’s position. PI, a constant quantitative anatomical parameter of the pelvis, is determined by the sum of PT and SS [18]. It is an objective measurement and plays a significant role in determining the sagittal balance of the spine. LL, which correlates with pelvic orientation, is crucial for postural alignment, and increased lordosis angles are closely related to a more horizontally inclined sacrum [19]. An earlier study has shown that a 1° pelvic reclination corresponds to approximately 0.7° functional anteversion of the cup during THA [14]. Hence, deviations in the orientation of the acetabular cup may influence the sagittal balance of the pelvis and spine, leading to alterations in PT, SS, and LL. In our study, we observed significantly higher values of standing and sitting SS values in the PG post-surgery. The variation in standing and sitting PT were significantly lower in the PG compared to the CG, while the variation in standing and sitting SS, as well as in LL were significantly higher. The evaluation of sagittal spinal balance is essential, and the SVA serves as a significant imaging parameter in this regard. Deviations in SVA can potentially heighten the risk of falls [20]. In our study, we found no statistically significant difference between the PG and CG in terms of the SVA and its variation. Therefore, it can be concluded that the body is capable of maintaining spinal sagittal balance by making compensatory adjustments in PT, SS, and LL after THA. The decrease in variation in standing and sitting PT was counterbalanced by the increase in variation in standing and sitting SS, as well as LL. However, these compensatory changes may contribute to the development of postoperative LBP.

An initial study has revealed a higher occurrence of chronic LBP in patients with lower SS, LL, and PI [21]. Furthermore, a systematic review encompassing 13 studies and a combined population of 796 patients with LBP and 926 healthy individuals found consistent evidence supporting a strong link between reduced LL and the presence of LBP [22]. Another radiographic study indicated that both hypo- or hyper-LL are associated with LBP and act as risk factors for lumbar degenerative disease [23]. These findings suggest a potential correlation between spinopelvic parameters and the development of chronic LBP. In this study, we observed that patients with postoperative LBP had significantly higher standing and sitting SS values. Additionally, these patients exhibited significantly increased variations in standing and sitting SS, as well as LL, compared to patients without postoperative LBP. Based on these subtle yet statistically significant variations, our observation suggests that the occurrence of postoperative LBP in patients with ONFH appears to be related to compensatory changes in SS and LL following surgery. Moreover, among patients experiencing postoperative LBP, the correlation analysis demonstrated a positive relationship between the variation in standing AI and the variation in standing PT, while it showed a negative correlation with the variations in standing SS and LL. This finding further supports the concept that the pelvis functions as a hinged structure, with the hip joint and the spine acting as its two ends. A smaller variation in the standing AI of the hip joint leads to compensatory increases in the variations in standing SS and LL in the spine.

This study has several limitations that should be acknowledged. Firstly, it was a single-center retrospective study, which may limit the generalizability of our findings to other populations. Secondly, the assessment of LBP symptoms was based on subjective reports, mainly derived from patient medical records or postoperative follow-up. This subjective approach makes it challenging to objectively quantify the severity of lumbar lesions. Thirdly, it should be noted that the lumbar spine encompasses various elements, including soft tissue, vertebrae, zygapophyseal and sacroiliac joints, intervertebral discs, and neurovascular structures, each susceptible to different stressors that may contribute to LBP [24]. Although, we rigorously controlled the enrollment conditions of patients and utilized postoperative lumbar MRI to mitigate interference from other causes of LBP, there remained certain limitations in the generalizability of our study’s findings.

## Conclusions

This study proposes a potential link between postoperative LBP and abnormal intraoperative cup orientation, characterized by reduced inclination and anteversion, following bilateral THA in patients with ONFH. Among those who experienced postoperative LBP, a positive correlation was found between the variation in standing AI and the variation in standing PT, while a negative correlation was observed with the variation in standing SS and LL. One possible mechanism behind this phenomenon is that a smaller intraoperative AI of THA, resulting from decreased inclination and anteversion, may lead to changes in pelvic tilt. Consequently, compensatory increases in standing SS and LL occur to maintain the SVA of the spine. These biomechanical alterations are implicated in the development of postoperative LBP.

## Data Availability

The datasets used and analysed during the current study available from the corresponding author on reasonable request.

## Abbreviations

LBP: Low back pain
THA: Total hip arthroplasty
ONFH: Osteonecrosis of the femoral head
PG: Pain group
CG: Control group
BMI: Body mass index
VAS: Visual analogue scale
AI: Ante-Inclination
SS: Sacrum slop
PT: Pelvic tilt
LL: Lumbar lordosis
MRI: Magnetic resonance imaging
ARCO: Association research circulation osseous
AP: Anteroposterior
CT: Computed tomography
CE: Center-Edge
AHI: Acetabular head index
PI: Pelvic incidence
SVA: Sagittal vertical axis
